# Telomere Biology, Erosion, and Age-Related Conditions: Insights from Down Syndrome and Other Telomere-Associated Disorders

**DOI:** 10.1007/s12035-025-05245-1

**Published:** 2025-08-01

**Authors:** Enikő Kutasi, Adina Chis, Mihaela Adela Vintan, Camelia AlKhzouz, Diana Alexandra Văduva, Andreea Cătană, Romana Vulturar

**Affiliations:** 1 Department of Molecular Sciences, Medical Genetics, “Iuliu Hațieganu” University of Medicine and Pharmacy, Cluj-Napoca, Romania; 2 Emergency Clinical Hospital for Children, 400370 Cluj-Napoca, Romania; 3 Department of Molecular Sciences, Cell and Molecular Biology, “Iuliu Hațieganu” University of Medicine and Pharmacy, Cluj-Napoca, Romania; 4 Department of Neurosciences, “Iuliu Hațieganu” University of Medicine and Pharmacy, Cluj-Napoca, Romania; 5 Department Mother and Child, “Iuliu Hațieganu” University of Medicine and Pharmacy, 400347 Cluj-Napoca, Romania; 6 Department of Medical Genetics, Regional Laboratory Cluj-Napoca, Regina Maria Health Network, Cluj-Napoca, Romania; 7 Department of Oncogenetics, Institute of Oncology “Prof. Dr. I. Chiricuță”, Cluj-Napoca, Romania

**Keywords:** Telomere length, Telomerase activity, Down syndrome, Premature aging, Age-related disorders

## Abstract

Telomeres play a crucial role in safeguarding DNA integrity. With each cell division, these protective structures undergo shortening, limiting the number of divisions to prevent improper genetic material distribution in aging cells. Senescent cells accumulate in tissues and contribute to age-related changes and decreased regeneration. Various genetic conditions are linked to premature aging and the early onset of age-related disorders. Down syndrome (DS), or chromosome 21 trisomy, is a relatively frequent aneuploidy, having an incidence of 1/1000–1/1100 newborns, and a major cause of intellectual disability. DS individuals exhibit a higher prevalence and earlier onset of age-related disorders, particularly Alzheimer’s disease, due to the buildup of beta-amyloid. In DS individuals, telomere erosion occurs at an accelerated rate, caused by the overexpression of numerous genes, and it is associated with various factors, including obesity, inflammation, hormonal fluctuations, physical or emotional stress, higher levels of reactive oxygen species, and autoimmune disorders. Although telomere length in DS children is initially higher than in the general population, their telomeres experience a more rapid shortening process. Developing strategies that target molecular pathways linked to telomere erosion and telomerase activity could become a key point for the therapeutic management of DS individuals.

## Introduction

Telomeres are ribonucleoprotein complexes spanning between 10,000 and 15,000 base pairs (bp) in size, with TTAGGG nucleotide repeats located at the ends of eukaryotic chromosomes representing non-coding DNA [1]. They are responsible mainly for maintaining chromosomal stability (i.e. preventing fusion between two chromosomes) and protection against degradation [2]. With each cell division, telomeres shorten due to the incomplete replication of linear DNA molecules, a phenomenon referred to as “the end-replication problem,” also being influenced by several factors such as oxidative stress or inflammation. Thus, their length can serve as a marker for cellular aging and senescence but also as indicators of individuals’ overall health status [1, 3].

The enzyme responsible for counteracting telomere shortening is called telomerase. It is active in the first weeks of embryonic development (up to 12 to 18 weeks of gestation), after that being silenced (with few exceptions, i.e., stem cells, germ cells, certain immune and endothelial cells), and thus having an impact on telomere length (TL) throughout life [4]. Telomeres prevent chromosome fusions, regulate gene expression, and have been linked to both tumor growth suppression and genomic aberrations leading to cancer initiation and progression [5, 6]. In cell populations where telomerase is active, it counteracts the loss of telomeric DNA by appending new TTAGGG sequences to significantly shortened telomeres. However, in most adult somatic cells, telomerase activity is suppressed. Consequently, telomeres naturally shorten with each cell division. This progressive shortening can compromise the regenerative capacity of tissues and is proposed as a characteristic hallmark of aging. Critically short telomeres trigger DNA damage response pathways and, if left unrepaired, it can lead to mitochondrial dysfunction, cellular aging, or cell death via apoptosis, eventually resulting in tissue or organ degeneration [1, 4–6]. As telomere erosion occurs as a natural process as we grow older, it contributes to a higher risk of developing age-related conditions [7].

Apart from telomere shortening, cellular senescence is influenced by various factors such as free radicals, toxins, and glycation, and it can be defined as the progressive accumulation of irreversible physiological modifications, leading to a higher susceptibility of organ degeneration [8]. Compared to the average shortening rate, accelerated telomere erosion plays an important part in the premature aging of the organism. Multiple studies highlight the role of telomerase inactivation and stress, especially chronic, in telomere erosion [9]. Thus, cellular senescence is primarily characterized by an irreversible growth arrest in dividing cells and a senescence-associated secretory phenotype (SASP) that occurs in both dividing and postmitotic cells. Importantly, telomere shortening is associated only with replicative senescence, whereas stress-induced premature senescence (SIPS) and oncogene-induced senescence (OIS) occur independently of telomere shortening [8, 9].

This systematic review summarizes the pathological pathways of accelerated telomere erosion and cellular aging, with a special focus on its occurrence in Down syndrome (DS) individuals. DS is the result of trisomy 21, and it is one of the most common causes of intellectual disability in both sexes. The incidence of DS is 1/1000–1/1100 in newborns [10]. While other early-onset age-related conditions have been reported in DS, early-onset dementia is the most common [11]. Even though it has been reported that telomere length is increased in children with DS compared with the general population, at adult age accelerated shortening was documented [11, 12]. Longer telomeres in DS children could be explained by having reduced cellular proliferation during embryonic development, as demonstrated by lower Ki67 and cyclin A levels in DS animal fetuses, compared to the control group [13], but they could also have multiple explanations such as advanced parental age at conception, comorbidities which slow down telomere shortening, such as hypothyroidism, autoimmune disorders, or hormonal imbalances, due to the lowered proliferation of lymphocytes, which might result in a protective action on telomere length [14]. While the connection between telomere length in peripheral blood cells and neurons is not well established yet, there are multiple studies stating rather contradictory results regarding telomere length in the brain or cerebellum of Alzheimer’s disease (AD) patients, compared to the control groups [15, 16], some even suggesting that the acquired shortening of telomeres can be linked to AD development, by incriminating vascular dysfunction or overall cellular senescence [17]. Potential mechanisms that can influence telomere dynamics in this population are the presence of metabolic syndrome, chronic oxidative stress [12], the overexpression of genes present on chromosome 21, such as the *APP* gene, or comorbidities [18].

The biological mechanisms underlying telomere shortening in DS are multifaceted. Telomerase is typically less active in somatic cells than in stem cells, leading to gradual telomere attrition over time [19, 20]. In the case of trisomy 21, the dysregulation of telomerase activity may exacerbate telomere shortening due to increased cellular proliferation and stress responses associated with the additional genetic material [21, 22]. Individuals with DS exhibit earlier onset of age-related phenotypes such as cognitive decline and physical health issues. Part of these complications is attributed to telomere dynamics, alongside oxidative stress or overexpression of the genes on chromosome 21, highlighting their importance in this population [19, 20].

Various therapeutic strategies are being explored to combat telomere shortening and its associated effects on aging. These include telomerase activators, which aim to enhance the activity of telomerase in somatic cells, potentially mitigating telomere attrition and promoting healthier aging [20, 22]. Additionally, tankyrase inhibitors are being investigated for their potential to stabilize telomeres by preventing their degradation during cell division. Other approaches involve the use of anti-inflammatory and antioxidative agents, which may help reduce cellular stress and inflammation that contribute to telomere shortening [19, 22]. Although no specific research has been published on maintaining telomere length in trisomy 21, by targeting these pathways, future approaches might address complications of Down syndrome related to telomere erosion, improving overall lifespan and quality of life for affected individuals.

## Methods

A comprehensive literature search was conducted accessing multiple electronic databases, including PubMed, Web of Science, and Scopus, to identify relevant articles analyzing the role of telomere erosion in early onset age-related conditions in individuals with Down syndrome. With a very few exceptions which were relevant for comparison of the described data, the search focused on articles published within the past 10 years, from January 2014 to May 2024, to ensure the inclusion of the most recent findings. The search strategy implied a combination of relevant medical subject headings (MeSH) terms and keywords, such as “telomere erosion,” “Down syndrome,” “age-related conditions,” and related synonyms. Boolean operators (AND, OR) were utilized to refine the search and ensure comprehensive coverage of the literature.

The inclusion criteria encompassed systematic reviews, meta-analyses, and original research articles that investigated telomere dynamics and their association with certain characteristics documented from individuals with Down syndrome, such as parental age, secondary diagnoses, or body mass index. To maintain the rigor of the review, only articles available in full text were considered eligible for inclusion, thereby minimizing the risk of bias introduced by incomplete access to data. The selection process involved scanning titles, abstracts, and full-text articles. Data extraction was performed systematically, focusing on key information including study design, participant characteristics, telomere measurements, and outcomes related to age-related conditions in Down syndrome.

## Overview of Telomere Biology and Factors Influencing Telomere Shortening

Telomeres are meant to protect chromosomes from losing genetic material during cell division. These structures are located at each chromosome end and contain thousands of TTAGGG hexanucleotidic repetitive sequences, which terminate with a G-rich single-stranded overhang of 100–200 nucleotide length [23].

**Fig. 1:**
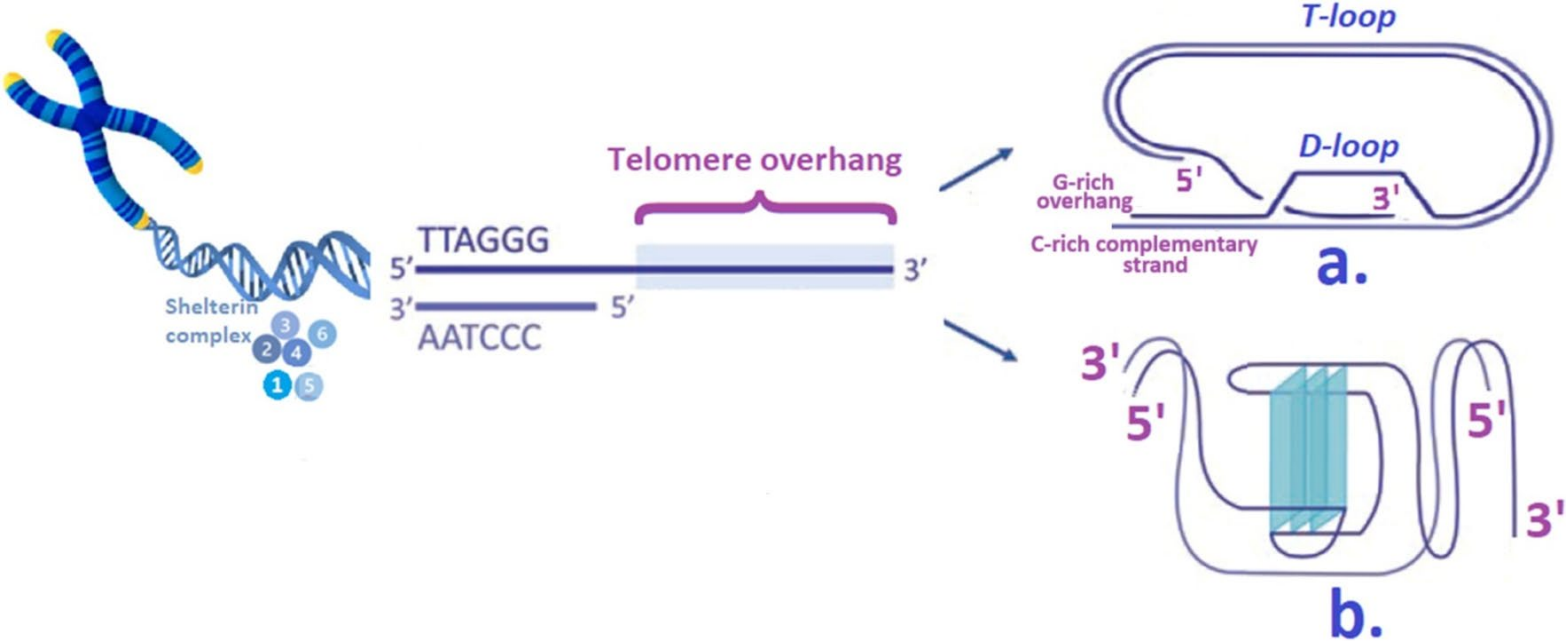
Telomere components with the 6 subunits of the shelterin complex (corresponding to TRF1, TRF2, POT1, RAP1, TPP1, and TIN2) [19–24]. Comprising the repetitive sequence of six nucleotides (5′-TTAGGG-3′) tandemly repeated over a span of 5–15 kilobases, human telomeres feature a G-rich strand that extends outwardly, forming a protrusion at the chromosome end known as the 3′ G-rich overhang [3, 29]. **a** The telomeric 3′ G-rich overhangs can fold back and invade the double-stranded telomeric DNA to form a displacement loop (D-loop), which facilitates the formation of the protective T-loops structure, which are akin to lariat-like structures (a structure resembling a lasso) [29, 30]. **b** Additionally, the 3′ G-rich overhang in telomeres has the capability to create stable intramolecular and intermolecular structures composed of four-stranded non-B DNA, known as G-quadruplex structures

The 3′ end of chromatin presents an extension called a 3′ G-rich overhang that forms a structure composed of 3 DNA strands as the overhang is tucked beneath the double-stranded telomeric DNA, forming a loop called D-loop, and it plays a crucial role in telomere integrity, preventing it from being recognized as damaged DNA and undergoing degradation (Fig. 1) [24]. The telomeric G-rich overhang is restricted to the 3′ telomere strand, whereas the 5′ end is inherently truncated during replication due to the end-replication problem, underscoring the asymmetric nature of telomere end processing [25].

The protein complex known to safeguard telomeres and thus maintain genomic stability is called shelterin, and it contains 6 subunits: telomere repeat-binding factor 1 and 2 (TRF1 and TRF2), protection of telomeres 1 (POT1) protein, repressor/activator protein 1 (RAP1), TRF1-interacting nuclear protein 2 (TIN2), and telomerase recruitment factor (TPP1). The TRF1 and TRF2 subunits of the shelterin protein complex bind to the double-stranded DNA of telomeres and prevent them from being recognized as double strand-breaks [26]. RAP1 binds to TRF2 and stabilizes the shelterin complex, while TIN2 connects TRFs. POT1 and TPP1 protect the 3′ single-stranded telomeric overhang, while the shelterin complex compacts telomeric chromatin, shielding it from damage and improper repair. It also facilitates telomere replication and maintains T-loop structures [27, 28]. Shelterin plays a pivotal role in regulating telomere length by suppressing pathways that trigger a local DNA damage response (DDR) and DNA recombination. By doing so, telomeres uphold cellular integrity and stability, serving as vital guardians of the genome [1]. In human chromosomes, telomeres adopt specific higher-order structures, such as T-loops and D-loops, to protect chromosomal ends from degradation and inappropriate DNA repair [1, 3].

In most somatic cells, with each division, telomeres shorten due to incomplete replication of the chromosomal ends (DNA replication machinery cannot replicate the last fragment of the lagging strand) (Fig. 1). Telomere shortening limits the number of mitotic cell divisions that can happen in almost each somatic cell; it leads to decreased proliferative potential and eventually senescence [31]. This process acts as a tumor suppressor mechanism, but it does not apply to male germ cells, where increased telomerase activity maintains telomere length for genome stability and prevents chromosomal segregation defects. Notably, telomere length increases in sperm as men age, and variation in telomere length among individual sperm cells likely reflects differences in early spermatogenic telomerase activity and/or varying oxidative stress levels during spermatogenesis [32].

As highlighted in the model proposed by Benetos et al. (2011), telomere shortening does not occur uniformly across all somatic cells; rather, it is predominantly observed in dividing (mitotic) cells, while postmitotic cells such as muscle cells and neurons maintain stable telomere lengths over time [33]. As tissues become older, mitotic somatic cells experience progressive telomere shortening, ultimately reaching their replicative limit (replicative senescence), known as the Hayflick limit [34].

Higher telomerase activity has been linked to preventing telomere shortening by capping; this process is an important factor in cancer cells [35]. Experiments in mice have shown that expanding embryonic stem cells in vitro can enhance telomere length. Hyper-long telomere mice exhibited reduced DNA damage, improved mitochondrial function, a younger metabolic profile, lower fat accumulation, better glucose and insulin sensitivity, and decreased levels of the senescence marker p21. They also had fewer spontaneous tumors compared to normal controls and experienced a 12.75% increase in median lifespan and an 8.4% increase in maximum lifespan [36, 37]. Although these might seem like exciting news, further research is needed to determine whether maintaining telomere length by various methods as a therapeutic measure, without increased cancer risk or other side effects, is achievable on a bigger scale [38, 39].

**Fig. 2:**
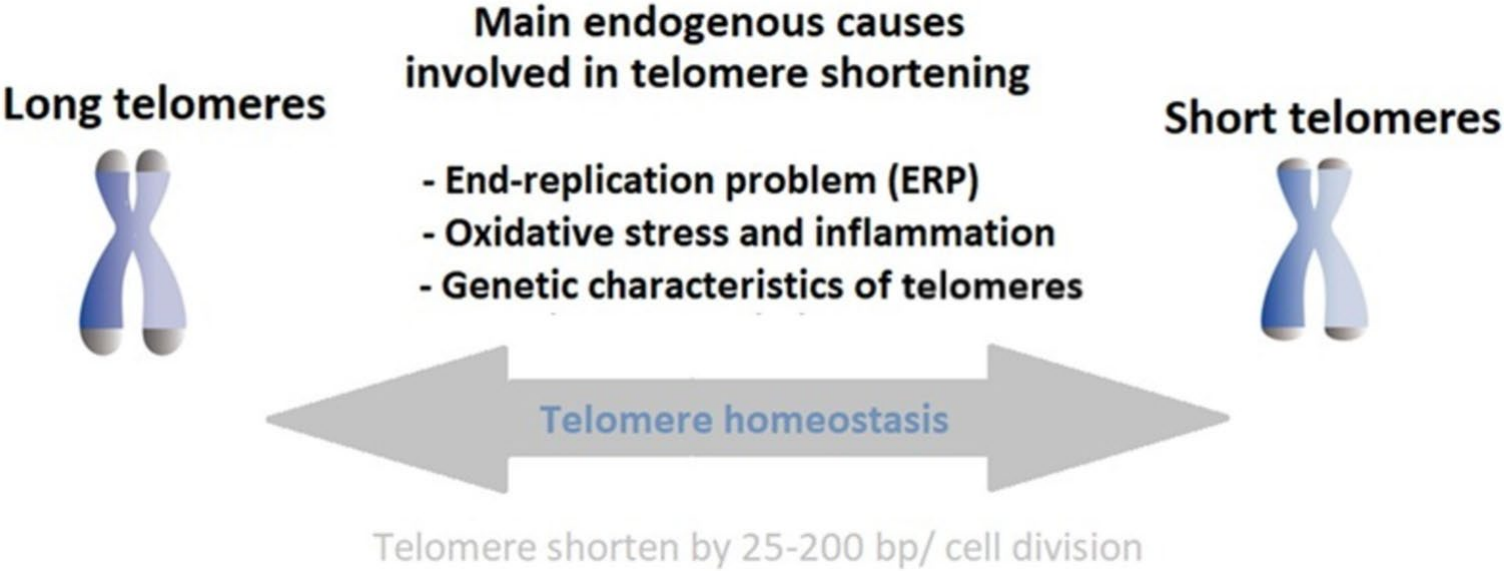
Telomere shortening process with each cell division; in some specific conditions, such as increased oxidative stress or certain cell types undergoing extensive division, this shortening can be even more pronounced [31, 32] sometimes exceeding 500 bp per cell division [40] (bp = base pairs)

Telomere length is influenced by multiple factors, including an important genetic component, the reported heritability ranging between 34% [41] and 82% [42]. In addition to genetic characteristics of telomeres, variations in telomerase genes also play a role in regulating the rate of telomere attrition and are associated with disease risk. For example, the single nucleotide polymorphism (SNP) rs2736100 in the *TERT* gene has been linked to telomere shortening, suggesting that the genetic variation of telomerase influences telomere length in the cell types where telomerase is active [43]. Additionally, alternatively spliced variants of telomerase reverse transcriptase may further regulate telomerase activity, and its role in aging and disease reveals the complexity of *TERT* regulation beyond its canonical function [44]. Despite many studies that support a significant contribution of heritability on TL, a recent study underlines that genetic factors can often overlap with environmental factors, making it difficult to precisely distinguish the contribution of each one (Fig. 2). The twin studies in which early life stress exposure is confounded with genetic factors support this idea [45]. Oxidative stress significantly contributes to telomere attrition through double-stranded DNA breaks and single-stranded damage caused by reactive oxygen species (ROS), which telomeric regions poorly repair, leading to incomplete replication of damaged DNA [46].

The End-replication problem (ERP) leading to progressive telomere shortening is the consequence of the inability to fully replicate linear chromosomal ends, the incomplete lagging strand synthesis, and the unidirectionality of DNA synthesis by conventional replicative DNA polymerase (from 5′ to 3′). During replication, DNA polymerase requires an RNA primer to initiate synthesis. DNA is replicated in two strands: the leading strand is synthesized continuously, while the lagging strand is made in short Okazaki fragments. After removing the RNA primers, there is no adjacent DNA segment to fill the final portion of the lagging strand, resulting in a slightly shorter newly replicated chromosome [47]. Telomerase can partially counteract the ERP in certain cell types, such as stem cells and germ cells. However, in most somatic cells, telomerase is not active to prevent telomere shortening over time [48].

The inheritance of genetic variations, in both telomeric and non-telomeric regions that contribute to telomere integrity, plays an important role in regulating TL [49]. Several genetic mutations can play a part in accelerated telomere attrition and developing telomeropathies:

- *DKC1* gene mutations leading to decreased telomerase activity. This mutation is incriminated in X-linked dyskeratosis congenita and it was one of the ideas supporting X-linked determinism of telomere length [50];
- *TERT* and *TERC* mutations also cause dyskeratosis congenita and lead to decreased telomerase activity and telomere shortening [51];
- Shelterin complex mutations lead to telomere instability [52];
- *RTEL1* mutations were also linked to dyskeratosis congenita and telomere shortening, as their role in preventing aberrant recombinations in telomeric DNA is affected [53];
- *PARN* gene mutations can lead to critically short telomeres. The *PARN* gene encodes an exoribonuclease involved in deadenylation, thus impacting the telomerase RNA component. *PARN* deficiency affects telomere length and stability and also downregulates the expression of shelterin transcripts [54];
- The *TCAB1* gene is crucial for proper telomerase assembly and transport, and thus its proper function [55].

Telomere length reprogramming in embryonic cells restores telomere length during early development, mainly through telomerase activity, with alternative mechanisms also contributing. This rejuvenation occurs during nuclear reprogramming, reversing epigenetic modifications from cellular differentiation [56]. Other studies support an additional genetic effect on TL given by the variability of the parental TL of the two gametes [57]. Moreover, there are studies that support a sex-specific heritability influence on TL, some findings pointing to a higher heritability from the mother [58]. There has been discussion about whether telomere length is X-linked, correlated with mitochondrial DNA, or influenced by parent-specific genetic imprinting, but evidence for maternal inheritance is inconsistent. Additionally, paternal age affects offspring telomere length [59]. Despite multiple divisions in sperm cells, TL seems to increase each year by approximately 57 bp, and higher paternal age leads to lengthened telomeres in the offspring [60]. Supporting the paternal influence on TL, studies have shown that a father affected by a genetic disorder associated with shorter telomeres will have children who present shorter telomeres compared to the general population, even if they are non-carrier [61]. On the other hand, telomere length does not seem to change in oocytes over time [62].

Numerous studies have shown that external factors like toxins, pollutants, diet, exercise, obesity, and stress impact TL. Psychological stressors, including abuse and major depressive disorder, are linked to shortened telomeres, while a balanced diet and regular exercise can help slow this shortening [63, 64].

**Fig. 3:**
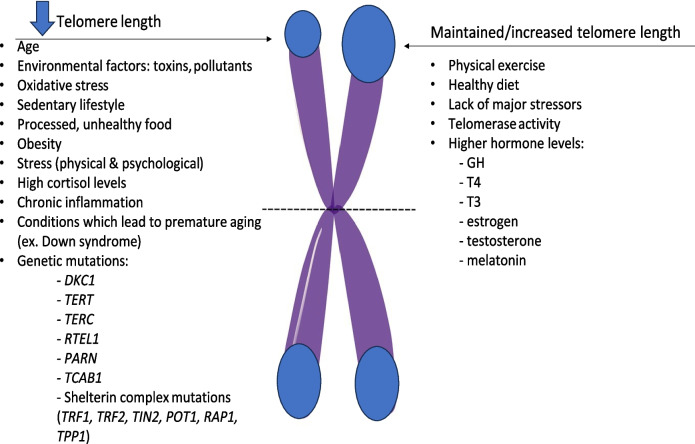
Factors involved in telomere length determination [38, 39, 41, 42, 45–64]. (GH, growth hormone; T4, thyroxine; T3, triiodothyronine)

Chronic inflammation present in various conditions can shorten telomeres due to an excess of reactive oxygen species (ROS). Hormonal factors have also been described to influence telomere length. While higher cortisol levels are associated with telomere damage and shortening, other hormones such as growth hormone (GH), insulin-like growth factor 1 (IGF1) thyroxine (T4), triiodothyronine (T3), estrogen, testosterone, or even melatonin can maintain telomere length [63, 64] (Fig. 3).

## Link Between Telomeres and Aging

The main features of the cellular aging process are genome instability, telomere attrition, oxidative stress and mitochondrial dysfunction, epigenetic alterations, loss of proteostasis, dysregulated nutrient-sensing, cellular senescence, stem cell exhaustion, and altered intercellular communication [65]. The main mechanisms for the involvement of telomeres in aging are limiting replicative capacity and triggering cellular senescence. Age-related diseases appear because of the decreased replicative capacity in tissues and organs due to the molecular events triggered by telomere shortening [66].

Cellular senescence is an irreversible process which causes cells to not re-enter the cell cycle and thus lose the ability to divide. Senescent cells remain metabolically active and can accumulate in tissues over time [7]. This leads to decreased regenerative capacity linked to aging, especially in tissues with high turnover rates, such as digestive tract mucosa, skin, and blood cells. This can be classified as a defense mechanism which protects the cells from acquiring further damage, and while the negative effect is potentially developing age-related diseases, cellular senescence also plays a positive role in tissue remodeling and in preventing tumorigenesis, thus being a tumor-suppressive mechanism. As mentioned in the introduction, senescent cells have the so-called SASP or “Senescence-Associated Secretory Phenotype” and produce more proteinases, cytokines, chemokines, and metalloproteases involved in proper wound healing. These cells are also known to escape apoptosis [7, 8, 49].

The impact of telomere shortening on the immune system deserves particular attention. Immune system cells are subjected to rapid proliferation, and their telomeres have high vulnerability to shortening. Telomerase activity also decreases in T-cells as time goes by, thus leading to even faster telomere shortening. In time, this can lead to improper immune activation when confronted with pathogens, affecting especially adaptive immunity. Furthermore, the combination of telomere shortening and rapid T-cell aging can lead to developing autoimmune conditions. Naive T-cells (those which have not yet encountered a pathogen) are affected in particular, leading to a reduced capacity to recognize new pathogens to the organism and thus reduced immune response. The reduced diversity of T-cells is also linked to the thymic involution happening with advanced age [23, 67, 68].

The accumulation of senescent T-cells leads to a state of chronic, low-grade inflammation, associated with a higher risk to develop age-related disorders. This state is known as inflammaging. Telomere shortening in T-cells has also been linked to a reduced response to vaccination; thus, the elderly may exhibit decreased antibody response to vaccination [69].

The early onset of age-related conditions has been reported in many genetic disorders, a few important ones being presented in Tables 1 and 2. Either hereditary and manifested from early childhood (Table 1), or acquired or adult-onset diseases (Table 2), these genetic disorders associate with premature aging. While accelerated telomere shortening is not the primary mechanism in the presented disorders, affected telomere maintenance or decreased telomerase activity can be observed in most of them, contributing to the decreased regenerative capacity and premature aging of the patients (see Tables 1 and 2) [50, 70–92].

**Table 1:** Hereditary genetic syndromes associating premature aging and telomere erosion [50, 70–87]

Syndrome	Manifestations	Affected genes	The molecular consequences on telomeres
Hutchinson-Gilford Progeria syndrome [70–72]	- Abnormal facies - Hair loss - Short height - Osteolysis - Limited joint mobility - Prominent veins - Sclerodermatous changes - Cardiovascular diseases	*LMNA*	- Alternative splicing of lamin A, producing a truncated protein called progerin - Accumulation of progerin into the nuclear envelope, disrupting its conformation and chromatin organization - Accelerated shortening of telomeres in fibroblasts
Werner syndrome [73–76]	- Gray, atrophic skin - Sclerodermatous features - Cataracts - Short stature - Thin limbs - High pitched/hoarse voice - Osteo-articular diseases - Stroke/neuropathies - Diabetes mellitus	*WRN*	- The absence of WRN helicase or presence of truncated WRN helicase affect the nascent lagging strand synthesis of telomeres, as well as the interaction with shelterin complexes, leading to defective telomere maintenance - Telomere shortening in skin and muscle cells, compared to the control group, especially as patients age - Mutated WRN helicase impact the DNA repair mechanisms, causing genomic instability
Dyskeratosis Congenita and other telomere biology disorders [50, 77]	- Bone marrow failure - Abnormal skin pigmentation - Nail dystrophy - Epiphora - Lung fibrosis - Liver cirrhosis - Osteoporosis - Leukoplakia - Immune deficiencies	*ACD, CTC1, DKC1*, *DCLRE1B, MDM4, NAF1, NHP2, NOP10, PARN, POT1, RTEL1, RPA1, STN1, TERT*, *TERC*, *TINF2, WRAP53, ZCCHC8*	Multiple molecular consequences, according to the affected gene: - Impaired telomerase recruitment to telomeres (*ACD*) - Impaired telomere replication (*CTC1*, *POT1*, *RTEL1, STN1*) and fragile telomere (*CTC1*) - Reduced TERC stability and activity (*DKC1, NAF1, NHP2, NOP10, PARN*) - General chromosome instability (*DCLRE1B)* - p53 activation and short telomeres (*MDM4*) - Defective telomerase regulation, impaired telomere stability (*RTEL1*) - Increased ssDNA binding affinity and increased telomere unfolding (*RPA1*) - Reduction of telomerase activity (*TERC, ZCCHC8*) - Reduction telomerase recruitment, processivity, and/or activity (*TERT*) - Multifactorial disruption of telomere maintenance (*TINF2*) - Impaired telomerase trafficking though Cajal body and recruitment to telomeres (*WRAP53*)
Ataxia–Telangiectasia syndrome [78, 79]	- Cerebellar ataxia - Oculocutaneous telangiectasias - Immune deficiencies - High cancer risk - Café-au-lait/hypopigmented macules - Neuropathy	*ATM*	- It seems that the inhibition of ATM protein induces telomere shortening in fibroblasts by a mechanism that involves interaction with the components of telomerase - The presence of truncated ATM protein induces a high degree of genomic instability
Lipodystrophy [80]	- Severe insulin resistance and diabetes mellitus - Dyslipidemia - Cardiovascular anomalies - Thinning skin, loss of subcutaneous adipose tissue - Risk for pancreatitis - NAFLD	*- LMNA* - *AGPAT2*, *PTRF*, *BSCL2*, *CAV1* genes (congenital generalized lipodystrophy) - *PPARG*, *AKT2*, *PLIN1* (familial partial lipodystrophy)	- Affected DNA repair - Telomere erosion in adipocytes, especially subcutaneous
Down syndrome [81]	- Intellectual disabilities - Speech and language challenges - Mongoloid facies - Protruding tongue - Short stature - Obesity - Higher risk for AD - Heart defects - Gastrointestinal anomalies (ex. celiac disease) - Immune deficiency - Hypothyroidism	Trisomy 21	- The complete mechanism involved in the high rate of telomere erosion is not fully understood, but it could imply an impaired stress mediated response, considering the localization of the dismutase gene (involved in the conversion of free radicals into peroxide) on the 21 chromosome
Rothmund–Thomson Syndrome [82]	- Poikilodermatous rash - Juvenile cataracts - Small stature-skeletal anomalies - Increased cancer risk	*RECQL4, ANAPC1*, *CRIPT, DNA2*	- The molecular roles of RECQL4 helicase on telomeres is not completely understood, but a direct interaction with telomere DNA, and also with shelterin subunits, was suggested - Mutations in the *RECQL4* gene were associated with telomere fragility and abnormalities - At the telomere level, DNA2 helicase is involved in the replication, mutations leading to decreased DNA replication
Bloom syndrome [83, 84]	- Growth deficiency - Facial dysmorphism - Skin hypersensitivity to UV irradiation - Early onset cancers - Immunological dysregulation	*BLM*	- BLM helicase is involved in telomere maintenance; it interacts with TRF2, promoting telomeric DNA synthesis - It is involved in recombination-dependent replication in human telomeres - Variability in the telomeric repeats of lymphoblasts
Cockayne syndrome [85, 86]	- Microcephaly - Growth delay - Senile appearance - Subcutaneous fat tissue atrophy	*ERCC6, ERCC8*	- Disrupted oxidative damage repair on DNA - CSB protein interacts with TRF2, thus the mutation influences telomere length and stability
Nijmegen Breakage syndrome [87]	- Dysmorphic facial features - Microcephaly - Growth delay - Intellectual disability - Immunodeficiency - Cancer risk	*NBS1*	- Disrupted maintenance of double-strand breaks and genome instability - Telomere shortening in leukocytes

*NAFLD* non-alcoholic fatty liver disease

**Table 2:** Acquired diseases or conditions associating telomere erosion and/or premature aging [88–92]

Disorder	Manifestations	Affected genes	The molecular consequences on telomeres
Aplastic anemia [88]	- Fatigue and weakness - Shortness of breath - Frequent infections - Easy bruising and bleeding	*TINF2, TERC, TERT, GATA2*	- High rate of telomere shortening, especially in the hematopoietic cells
Idiopathic pulmonary fibrosis [89]	- Chronic dry cough - Shortness of breath - Weight loss - Finger clubbing	*TERT, TERC, SFTPC, MUC5B*	- Accelerated telomere shortening - Chromosomal instability - Lower telomerase activity in the fibrotic tissue
Cancers [90]	- Weight loss - Fatigue - Loss of appetite - Manifestations characteristic for the cancer site	*TP53, BRCA1, BRCA2, KRAS, NRAS, APC*, etc	- Although not commonly encountered in cancer tissues, telomere shortening can facilitate malignant transformation (by inducing chromosomal instability and influencing tumor cell plasticity)
Chronic stress and inflammation [91, 92]	- Chronic fatigue - Weakened immune system - Increased heart rate and blood pressure etc	*NR3C1, IL6, CRHR1*	- Changes in telomere structure - Accelerated telomere shortening - Childhood stress has a very important effect on telomere shortening, which, so far, was mostly studied in PBMCs

*PBMCs* peripheral blood mononuclear cells

## Gene Overexpression and Early-Onset Age-Related Manifestations in Down Syndrome

Down syndrome is caused by the presence of an extra copy of chromosome 21, which usually appears due to the meiotic nondisjunction of chromosome 21. Mosaic DS is a result of a mitotic nondisjunction, which happens after fertilization, and thus, some cellular lines will have an extra chromosome 21, while the others have 46 chromosomes. This form associates with milder features and less severe intellectual disability [93, 94].

Another mechanism which can lead to DS in the offspring is the presence of a Robertsonian translocation involving chromosome 21. Robertsonian translocation means the fusion of two acrocentric chromosomes, and while being a carrier will not impact the parents’ phenotype, it increases the risk of having children with DS or fertility issues. If the Robertsonian translocation appears between two chromosomes 21, the risk for trisomy 21 is 50%, as the other 50% of the potential offsprings will present monosomy 21, a condition that is not compatible with life. So, when a parent carries a 21–21 Robertsonian translocation, virtually, all live births of his offsprings will result in a child with Down syndrome [95, 96].

The life expectancy for these individuals is up to their 60 s or even more with proper care. The quality of life for DS individuals highly depends on the severity of the clinical manifestations and their access to proper healthcare, as well as special education and occupational therapy [97–99].

The presence of an extra chromosome 21 leads to several disrupted cellular pathways, such as glutamatergic and GABAergic transmission in the nervous system, increased oxidative stress and inflammation, and mitochondrial dysfunction [100]. Neuroinflammation is another process which might lead to a difference in cellular pathways associated with trisomy 21 [97]. Gene expression is also negatively influenced by trisomy 21 [101]. There are 233 coding genes on chromosome 21; in addition, about 1050 long non-coding RNAs (lncRNAs) and 30 microRNAs (miRNAs) were identified, molecules that regulate gene transcription, suggesting that other candidate genes or regulatory regions encoded on chromosome 21 may be responsible for a phenotypic picture characteristic for DS individuals [102–104]. In this line, it was suggested that more than 600 genes in the whole genome are overexpressed because of the presence of an extra chromosome 21 in DS [105]. Therefore, some studies have shown that different disomic genes present a variable expression pattern in tissues with trisomy 21, suggesting a higher instability in the control of the transcription process [106]. Moreover, a study conducted on discordant monozygotic DS twins has identified, in induced pluripotent stem cells (iPSCs) derived from fibroblasts, some regions called gene expression dysregulated domains; they suggest that these domains can appear due to the overexpression of some genes located on chromosome 21, leading to important chromatin modifications and being an important factor in the development of [107, 108].

The most important genes on chromosome 21 overexpressed in trisomy 21 and contributing to the early onset of age-related conditions are described in Table 3 and discussed below [109].

**Table 3:** Genes located on chromosome 21, overexpressed in trisomy 21 with an important role in early-onset age-related manifestations affecting DS individuals [110–116]

Genes	Molecular mechanisms	Phenotypic correlations
*APP* [110, 111]	Accumulation of beta-amyloid Increased rate of apoptosis in glial cells, and enhances p53-mediated apoptosis	Predisposition to AD
*DSCR1/RCAN1* [111]	Dysregulation of calcineurin = > altered calcium signaling	Altered cardiac development Immune deficiency
*DYRK1A* [112]	Cell cycle dysregulation Altered neuron development	Cognitive impairment
*ETS2* [112]	Increased rate of apoptosis in multiple cells, including neurons Increased expression of p53	Neurodegeneration Smaller thymus Abnormal lymphocytes Skeletal anomalies Potential tumorigenesis
*CBS* [113]	Altered homocysteine metabolism Increased rate of apoptosis, which may contribute to the neurodegeneration	Cardiovascular and neurological disorders
*SOD1* [114, 115]	Overproduction of peroxides	Telomere shortening due to higher oxidative stress and an imbalance with compensatory antioxidants
*OLIG2* [116]		Altered oligodendrocytes development
*SIM2* [117]	Dysregulation of gene expression	Altered neural development and cognitive function
*SON* [111]	Altered transcription and splicing	
*ITSN1* [118]	Altered endocytosis and signal transduction	Altered cellular communication and synaptic function
*RUNX1* [111, 112]	Influences hematopoiesis and angiogenesis	Altered hematopoiesis, influencing cell proliferation, prognostic marker in cancer, risk of developing leukemia

Individuals with DS face a significantly heightened risk of developing AD at an early age, several genes contributing to the phenotypes associated with this condition. The amyloid precursor protein (*APP)* gene has long been recognized as a primary contributor to this risk due to its role in amyloid-β production, which aggregates to form plaques characteristic of AD pathology. Recent research highlights the importance of other genes on chromosome 21 that may also influence AD pathology. For instance, *DYRK1A*, which encodes a kinase involved in tau phosphorylation, has been implicated in promoting neurofibrillary tangles, another hallmark of AD [119]. Additionally, *RCAN1* (regulator of calcineurin 1) may influence neurodegeneration pathways, further complicating the genetic landscape affecting cognitive decline in this population [120]. Genes such as *DSCAM*, *S100B*, *BACE2*, and *TIAM1* have been implicated in modulating various phenotypes associated with both DS and AD. The protein DSCAM (Down syndrome cell adhesion molecule that belongs to the immunoglobulin superfamily) is involved in synaptic function and in neuronal development, which may affect cognitive decline. S100B is a calcium-binding protein that can influence neuroinflammation and neuronal survival, while BACE2 is thought to play a role in amyloid processing and clearance and is potentially protective against amyloid-beta accumulation. TIAM1, a guanine nucleotide exchange factor, has been linked to neuronal signaling pathways that could impact neurodegeneration. The interplay among these genes underscores the complexity of DS and its association with AD, suggesting that therapeutic strategies targeting multiple genetic pathways may be necessary for effective intervention in this population, as discussed in the book “Genetics and Neurobiology of Down Syndrome” [119]. Thus, while APP remains a central player in the relationship between DS and AD, it is obvious that other genes on chromosome 21 significantly influence disease progression and cognitive outcomes. This intricate interplay suggests that therapeutic strategies targeting multiple genes or pathways may be necessary to effectively address the heightened risk of AD in individuals with DS. Continued research into these biological factors holds promise for developing interventions aimed at mitigating cognitive decline and improving quality of life for affected individuals.

While it is not located on chromosome 21, the *hTERC* gene is reported to have additional copies in a higher number of cells in individuals with a supernumerary chromosome 21 [121]. The exact mechanism for this is yet to be studied, but it might be linked to the altered overall gene expression which can appear due to the excess genetic material. *hTERC* encodes a telomerase subunit and might be associated with higher telomerase activity, as shown in endometriosis [122] and bovine blastocysts [123]. However, excessive telomerase activity might also be responsible for neurodegenerative processes [121, 124]. Telomerase activity is also enhanced by higher telomerase reverse transcriptase levels, maintaining TL by adding repetitive DNA sequences to the ends of chromosomes, compensating for the loss of DNA during replication and helping cells avoid senescence [124]. The involvement of telomeres in the pathogenesis of neurodegenerative disorders is still a controversial topic in the scientific community. While it is sustained by studies [125–127] that have shown a higher rate of telomere shortening in the PBMCs of patients with Alzheimer’s, Parkinson’s, and Huntington’s diseases, compared to healthy individuals [125–127], other studies show no telomere shortening in blood cells of patients with neurodegenerative disorders. Moreover, studies showed that *TERT* gene polymorphisms can increase the risk of neurodegenerative disorders in humans, emphasizing a possible link between telomeres/telomerase and the risk for developing these conditions [128]. Although telomere shortening was only analyzed and documented in PBMCs, and not in postmitotic cells, such as neurons, thus not having a direct correlation with neurodegenerative disorders, it can serve as a marker for generally increased oxidative stress which will result in telomere erosion and dysfunction or senescence in different cell types [129, 130]. Notably, in the brain, TERT primarily exerts non-telomeric functions, including regulation of gene expression, synaptic signaling, and neurotrophic support [131]. In postmitotic cells such as neurons, which do not divide and thus do not shorten their telomeres, telomerase activity is absent from the nucleus, making TERT impact telomere length. Nevertheless, neuronal telomeres can accumulate DNA damage over time, leading to a senescence-like phenotype that is independent of telomere shortening and cannot be counteracted by telomerase [131]. Interestingly, restoring physiological levels of TERT in neurons has been shown to enhance synaptic function and cognitive performance through transcriptional mechanisms rather than telomere elongation. Furthermore, pharmacological activation of TERT in aging tissues ameliorates multiple aging hallmarks—including DNA methylation changes and cellular senescence—through epigenetic regulation, again independently of telomere length [132].

There is also an increased expression of genes encoding interferon (IFN) receptors on chromosome 21 [133]. Some of the neurological features described in DS might be linked to the overactivation of the IFN pathway. IFN hyperactivity leads to accumulation of tryptophan catabolites, which involves neurotoxicity [134]. The increased expression of interferon-related genes creates an inflammatory feedback loop in which overexpression of IFN leads to its further production. This may also lead to abnormal activation of macrophages and dendritic cells, as IFN is responsible for this process [133, 135, 136].

Interferon hyperactivity, as observed in conditions like DS, can lead to sustained activation of the JAK/STAT pathway. This prolonged signaling may contribute to chronic inflammation and immune dysregulation. The HIF pathway is activated in response to hypoxia (low oxygen levels) and plays a role in cellular adaptation to oxygen availability. Interferons, particularly interferon alpha (IFN-α), can influence HIF pathway activation. Interferon-induced activation of the HIF pathway can have various implications, including the modulation of immune responses, angiogenesis, and metabolic adaptation [137, 138].

Altered expression of different types of non-coding RNA has also been reported in trisomy 21. While non-coding RNAs do not directly lead to protein synthesis, they have a crucial role in post-transcriptional regulation of gene expression. These RNAs can also impact telomerase activity and thus telomere maintenance, as well as cellular microenvironment, contributing to cellular senescence and overall aging [139–141].

**Fig. 4:**
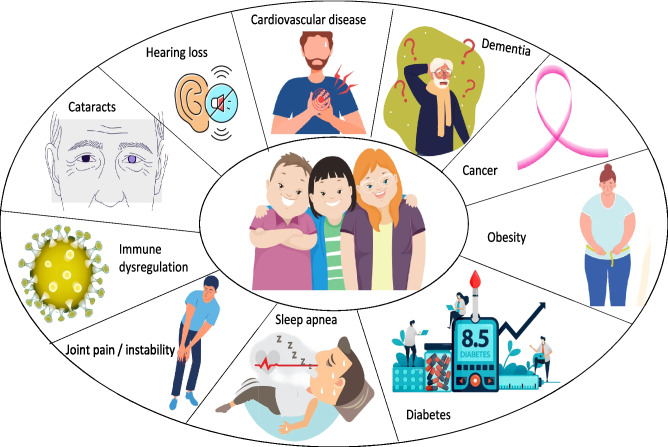
Age-related conditions in Trisomy 21 [11, 12, 97, 142–144]

Several age-related conditions were reported to affect DS individuals earlier and with higher prevalence compared to the general population (Fig. 4) [11, 12, 97, 142–144].

While DS patients may present different types of early onset dementia, AD is the most common form described [104]. As indicated in Table 3, the main shared factor between DS and AD is overexpression of the *APP* gene (located on chromosome 21) that encodes the precursor of Aβ-peptide found in the amyloid plaques present in the brain of AD patients [145, 146]. The overexpression of the *APP* gene can also be attributed to the presence on chromosome 21 of a transcription factor encoder, EST2, that activates the *APP* gene via promoter [147]. Besides these overexpressed genes, neuroinflammation is another important contributor to the pathogenesis of both DS and AD. In patients with DS, the inflammatory response can be attributed to the overexpression of the astrocyte-derived *S100B* gene (located on chromosome 21) and neuroinflammatory cytokine *IL-1β* gene (observed as an early event in DS) [148]. In AD, neuroinflammation appears because of activated microglia and astroglial cells that surround the amyloid plaques; the activation of microglia is explained by the overproduction of an α-secretase cleaved fragment (because of APP gene overexpression and activation of the non-amyloidogenic pathway). In turn, activated microglia induce the overexpression of *IL-1β* and *S100B*, influencing the pathogenesis of both pathologies [104]. This neuroinflammatory state and peripheral inflammation that is also present in these pathologies may be responsible for an accelerated division of leukocytes and in turn for telomere shortening [149, 150]. In addition to late-onset AD (majority cases), about 5% of AD cases present clinical symptoms before the age of 65, which is considered to be early-onset AD (EOAD). Although in most EOAD cases, the causality cannot be explained; about 5% are linked to mutations in genes located on chromosome 21, such as *APP* or *PS1/2* genes. Considering that trisomy 21 individuals represent the largest cohort of EOAD patients, this pathology is considered the leading genetic risk factor for EOAD [104]. Acquired agnosia and apraxia have been described in DS patients at ages as early as 30 years. Other mental health issues to consider include depression and anxiety symptoms which may appear, especially as patients grow older [81, 110, 111].

Obesity is described with higher prevalence as well. There are multiple possible explanations for this phenomenon, for example, basal metabolic rate can be lower and behavioral habits may include unbalanced diet and lack of physical activity or secondary medical conditions such as hypothyroidism. Physical activity is also influenced by the hypotonia and the poorer motor skills and coordination these patients exhibit [144, 151].

Heart disease, arterial hypertension, diabetes mellitus, particularly type II, and sleep apnea have also been described in these patients [152, 153].

Gastrointestinal symptoms, besides the higher risk for celiac disease, may include constipation and gastro-esophageal reflux. DS individuals can also present low immune response to hepatitis A and B, highlighting the need for immunization [154].

Early onset cataracts and hearing impairment are common concerns as well in trisomy 21. Musculo-skeletal issues, such as joint pain and instability and osteoporosis, also arise with higher prevalence and earlier than in the general population [155–157].

The higher risk of developing these disorders, many of them being multifactorial, points to the importance of regular health screenings in DS individuals and also of early lifestyle interventions, which may limit the negative impact on the individuals’ health status.

## Telomere Shortening in Down Syndrome Individuals

Gruszecka et al. report longer average telomere length in the leukocytes of DS children and a young adult group they analyzed (ages 2–21 years, mean age 4.5 ± 3.1), comparing to the control group (ages 2–17 years, mean age 4.5 ± 3.4). The average TL was 50.46 arbitrary units (au) in the DS group, and 40.56 (au) in the control group (*p* = 0.0026). There was no significant difference in the TL depending on the sex of the individuals in either of the groups. In this study, telomere shortening was observed as probands advanced in age, but without statistical significance [14].

The mean TL in DS children was significantly higher than in the control group, regardless of the age of the parents [14]. An accelerated telomere erosion was observed by Horvath et al. in blood and brain tissue of DS subjects, but not buccal mucosa. However, the number of both DS individuals and the members of the control group in this study were limited, but the data was consistent in several determinations [12].

Immune system modifications, either immunodeficiency or autoimmune disorders, can also affect cellular metabolism and thus telomere length [158].

Telomere erosion rates were reported to be 58 bp/year in DS individuals, compared to 38 bp/year in control groups by Bhaumik et al. [159]. According to this study, the difference in TL between the DS group and euploid children is about 400 bps in newborns, in favor of the DS group. However, in older children, the difference drops to 150 bps, and at the age of 8, it drops below the TL mean of healthy children [159]. It is worth mentioning that in a previous study conducted by Vaziri et al., the TL shortening rate was reported to be 133 bp/year in DS individuals and only 43 bp/year in euploids [160].

Abdel-Salam et al. report higher telomerase activity in the lymphocytes of a group of DS children aged 2 to 9 years (mean 4.2 ± 1 year), compared to an age-matched group of children without chromosomal aneuploidies [161]. They also report increased levels of apoptosis markers, such as members of the tumor necrosis factor receptor family, and decreased levels of antiapoptotic protein Bcl-2 in DS individuals compared to control groups [161]. We did not find studies on telomerase activity in newborns with DS, or in children older than 9, in order to properly link telomere dynamics in DS individuals to the observed telomerase activity.

During early embryogenesis, telomere length is rapidly restored through a biphasic process: initially via the alternative lengthening of telomeres (ALT) pathway, independent of telomerase and based on recombination, and later through telomerase activation at the blastocyst stage. This transition ensures genomic stability and offsets telomere shortening inherited from aged gametes [162]. Notably, shortened maternal telomeres have been associated with an increased risk of trisomy 21. For instance, a study conducted by Albizua et al. found significantly shorter telomeres in mothers of children with DS, particularly in cases involving nondisjunction during meiosis II, pointing to a potential link between TL, reproductive aging, and chromosomal segregation errors [163]. To determine in which meiotic division the disjunction error happened, over 1500 SNPs specific to chromosome 21 were tested to define the regions of recombination. In the nondisjunctions happening in meiosis I, a single recombination was observed in the telomeric region, while in those happening in meiosis II, the recombination was observed in the pericentromeric region [164]. In contrast to continuously dividing somatic cells such as blood cells, which undergo progressive telomere shortening, oocytes exhibit unique telomere dynamics, including minimal telomerase activity and the use of alternative lengthening of telomeres (ALT) mechanisms during early development, helping preserve or restore telomere length across generations [162].

More recently, another case–control study has shown that the maternal peripheral blood TL was significantly shorter in the trisomy 21 group compared with the control group [165]. Moreover, it found a significant positive correlation between the maternal and fetal TL in the trisomy 21 group; these results suggest that TL shortening could be responsible for the nondisjunction of chromosome 21 during meiosis and therefore for trisomy 21 pregnancies [165]. To assess the influence of environmental and genetic factors on TL shortening, the same authors have investigated the effect of maternal age, occupation, and nationality on maternal TL; even if there was a tendency of TL shortening in the advanced age group, opposite to the Albizua study, the overall correlations were not significant. Finally, to identify any predictive role of TL shortening for trisomy 21 pregnancies, the study investigated the relation between different parameters (i.e., maternal age, maternal lymphocyte TL, occupation, nationality) and trisomy 21; it found that shortened lymphocyte TL in the mothers was a predictive factor for trisomy 21 in the offspring with an accuracy of 66.5%. When the study tested maternal age and maternal lymphocyte TL shortening taken together as predictive factors for trisomy 21 in the offspring, this accuracy increased up to 80.8% [165]. Taken together, these studies highlight a possible predictive risk for trisomy 21, although new studies are necessary for identifying the variability of TL at birth, as well as how environmental factors influence the TL shortening in general, and in DS specifically.

However, in paternally originated DS cases (meaning the chromosomal nondisjunction happened in the paternal gamet), no difference was reported regarding the parents’ mean TL compared to that of parents of children from the control group [80]. This suggests that maternally originated trisomies (where the chromosomal nondisjunction happens in the maternal gamet) are more likely due to quicker molecular aging, while the paternally originated trisomies are probably only sporadic accidents [81, 160, 165].

Regarding the telomerase activity in young DS individuals, this is increased in the peripheral blood cells, compared to healthy controls, which is associated with longer telomeres observed in children with DS. This elevated activity may be influenced by factors such as *ETS-2* gene overexpression and hormonal changes like increased estradiol levels. With aging, DS individuals experience accelerated telomere shortening. Although telomerase activity is initially high, it does not prevent telomere attrition over time. Telomere length in the leukocytes of individuals with DS shortens significantly, especially beyond 40 years of age, where a rapid telomere attrition of about 47% has been reported [14].

## Future Directions and Potential Therapeutic Measures for Accelerated Telomere Shortening

Exploring potential therapeutic interventions to prevent rapid telomere erosion in DS is a promising topic for research in this field. Understanding the molecular mechanisms involved in telomere erosion, particularly in the context of trisomy 21, may open access to targeted interventions. Monitoring telomere length in PBMCs as part of regular health assessments might be beneficial, in addition to promoting preventive measures, such as healthy dietary habits, or regular physical activity, into routine healthcare for individuals with DS.

One potential therapeutic focus is on enhancing telomerase activity, the enzyme responsible for maintaining telomere length. Several studies have shown that some substances can act like telomerase activators, leading to telomere extension. For instance, two different compounds derived from *Astragalus membranaceus* (traditionally used in Chinese medicine), TA-65 and cycloastragenol, were tested as telomerase activators, the obtained results being promising [14, 166, 167]. Recent studies have drawn attention to the non-telomeric effects of telomerase induction, highlighting its therapeutic relevance in neurodegenerative disorders such as AD and Parkinson’s disease (PD). Beyond its classical role in telomere elongation, TERT has demonstrated multiple neuroprotective functions. In PD models, TERT activation enhances autophagy, reduces α-synuclein accumulation, and improves motor performance—effects independent of telomere maintenance [168]. Similarly, pharmacological activation of telomerase in hippocampal neurons exposed to amyloid-β has been shown to increase the expression of neurotrophic and synaptic plasticity-related genes, indicating a potential for cognitive protection in AD [169]. Supporting this, overexpression of TERT in AD mouse models preserved neuronal integrity and attenuated cognitive decline through mechanisms not directly linked to telomere length [131]. On a molecular level, TERT activation has also been associated with remodeling of DNA methylation landscapes and mitigation of several hallmarks of aging—such as inflammation, mitochondrial dysfunction, and cellular senescence—further underscoring its non-telomeric, pleiotropic actions in age-related neurodegeneration [132]. Collectively, these findings position TERT induction as a promising multifaceted strategy with translational potential for modifying the progression of AD and PD. Another tested approach for telomerase activation was gene therapy. Thus, Bernardes de Jesus et al. have shown a significant reduction of aging markers in mice, without increasing cancer incidence, using a viral vector to express *TERT* [170]. Due to ethical concerns, as well as the unknown long-term effects, telomerase gene therapy has not yet been tested in humans. However, in the future, gene therapy can prove to be a useful tool in reversing or stopping the aging process, but also for disease treatment [22].

Additionally, lifestyle and pharmacological interventions may be explored. Lifestyle changes, such as adopting a balanced diet, regular exercise, and stress management, could positively impact overall health and potentially influence telomere dynamics. Research into pharmacological agents that target pathways related to telomere maintenance may provide novel therapeutic options.

Antioxidants, such as vitamin C, E, or polyphenols, were proven to have a protective effect against telomere erosion, due to their effect of neutralizing free radicals that can affect DNA stability [22]. Moreover, through mechanisms which involve reducing oxidative stress and inflammation, anti-inflammatory substances such as omega-3 fatty acids are potential pharmacological approaches to slow down telomere erosion [22]. Personalized interventions that address specific risk factors and support telomere health could be a promising approach.

However, one of the most important aspects to consider is the potential side effects of any therapeutic measure that might be explored for slowing down telomere erosion rates in DS. Further research is much needed to understand the intricate mechanisms of molecular aging and its clinical consequences in individuals diagnosed with trisomy 21 and other disorders which lead to premature aging.

## Conclusions

Telomeres are structures which protect the integrity of DNA. With each cell division, telomeres become shorter, and thus, they only allow a limited number of cell divisions to prevent the inadequate distribution of genetic material in older cells.

For over three decades now, significant progress has been made in understanding the essential role of telomeres in cell homeostasis and aging. Research on model organisms has greatly expanded our knowledge of telomere biology, revealing the complex mechanisms cells use to maintain telomere length. These findings outline the evolutionary importance of telomere structure conservation. Senescent cells build up in tissues, leading to age-related modifications and lower regenerative capacity.

Several genetic conditions were linked to premature aging and early development of age-related manifestations. Down syndrome, or trisomy 21, is one of the most frequent aneuploidies diagnosed and one of the leading causes of intellectual disability in both sexes. Several age-related manifestations were reported to appear with higher prevalence and earlier in DS individuals, the most striking being dementia and, more specifically, AD.

Telomere erosion appears with a higher rate in DS individuals, due to the overexpression of multiple genes situated on chromosome 21, but also other genes, the expression of which seems to be influenced by the extra copy of chromosome 21. While telomere length in newborns and children under the age of 8 is higher in DS groups than in the general population, the shortening of telomeres happens with a significantly higher rate in DS individuals.

Obesity, inflammation, hormonal levels, physical or emotional stress, higher levels of reactive oxygen species and peroxides, autoimmune disorders, etc., could all lead to accelerated telomere erosion. Defects or depletion of telomere components can determine cell fate, often leading to age-related conditions, senescence, or cell death. Thus, an individual’s TL reflects the interactions between genetic and environmental factors. This has spurred a new field of research in telomere biology regarding the impact of lifestyle changes and potential therapeutic measures which target either telomerase or factors leading to telomere erosion. DNA damage accumulation and short telomeres in senescent cells significantly impact lifespan and health of both the cells and the organism as a whole.

Targeting molecular pathways involved in telomere erosion and telomerase activity could be part of the therapeutic management of DS individuals, and thus, further research is needed in this field to understand more about the mechanisms involved in premature aging and age-related conditions.

## Data Availability

No datasets were generated or analysed during the current study.
